# Sevoflurane relieves hepatic ischemia–reperfusion injury by inhibiting the expression of Grp78

**DOI:** 10.1042/BSR20180549

**Published:** 2018-10-05

**Authors:** Di Liu, Xin Jin, Chunqi Zhang, You Shang

**Affiliations:** Department of Anesthesiology, The First Affiliated Hospital of Jinzhou Medical University, Jinzhou 121000, China

**Keywords:** apoptosis, Grp78, hepatic ischemia-reperfusion injury, Sevoflurane

## Abstract

**Purpose:** This article aimed to study the role of sevoflurane pre-conditioning in hepatic ischemia–reperfusion and its potential mechanism.

**Methods:** Rat liver ischemia–reperfusion model was constructed. Serum TNF-α, IL-1β, IL-10, and IL-6 concentrations were detected by ELISA. Malondialdehyde (MDA), superoxide dismutase (SOD), and nitric oxide (NO) in liver homogenate were determined. Hematoxylin–Eosin (HE) staining, Tunel, and immunohistochemistry were performed. Ischemia–reperfusion hepatocyte model was established. Cells transfection was conducted. Apoptosis was observed by flow cytometry. Quantitative real-time PCR (qRT-PCR) and Western blotting analysis were used.

**Results:** Compared with I/R group, liver damage degree, liver cell apoptosis, and glucose regulatory protein 78 (Grp78) expression was obviously reduced in rats of SEV group. TNF-α, IL-1β, and IL-6 concentrations were also significantly increased (*P*<0.01). MDA and NO concentrations were dramatically lower (*P*<0.01) and SOD concentration was significantly higher (*P*<0.01). Apoptosis rate, Grp78, PERK, eIF2α, and p-c-JNK/JNK expression was also significantly decreased (*P*<0.01). Sevoflurane significantly reduced apoptosis and expression of PERK, eIF2α, p-c-JNK/JNK by inhibiting the expression of Grp78 (*P*<0.01).

**Conclusion:** Sevoflurane relieves hepatic ischemia–reperfusion injury by inhibiting the expression of Grp78.

## Introduction

Ischemic reperfusion is a necessary part of liver surgery or liver transplantation. Inevitable hepatocellular damage can be caused during ischemia process and liver function is also unlikely to quickly return to normal after reperfusion [1]. In most circumstances, liver inflammation, damage, and even severe liver dysfunction would be occurred after surgery [2]. Severe liver ischemia–reperfusion injury can lead to multiple organ failure and even death, which is one of the key factors that cause severe adverse prognosis [3]. Thus, prevention and alleviation of hepatic ischemia–reperfusion injury have an important impact on improving patients’ prognosis and reducing their mortality. Complex physiological processes were involved in hepatic ischemia–reperfusion injury, such as metabolic disorders, oxidative stress, and inflammatory response [4,5]. Discovery of the exact mechanism leading to hepatic ischemia–reperfusion injury was important to improve patients’ prognosis.

Accumulated studies have found that the abnormal expression of genes has an important influence on various diseases occurrence and development [6,7]. Therefore, the discovery of the exact molecular biomarker is very important which can provide the exact therapeutic target for these diseases. Glucose regulatory protein 78 (Grp78) is a multifunctional protein [8]. Grp78 is a contributing factor for the invasion and metastasis of hepatocellular carcinoma, and its role in ischemia–reperfusion injury has also been a concern of researchers [9,10]. Previous researches have shown that Grp78 expression was up-regulated in the ischemia–reperfusion experiments of kidney, brain, and retina [11–13]. These findings provide a new idea for the treatment of ischemic reperfusion diseases. That is, the prognosis of ischemic reperfusion diseases can be improved by inhibiting the expression of endogenous GRP78. However, the effect of GRP78 in hepatic ischemia–reperfusion still needs further investigation. As an inhalation anesthetic, sevoflurane has been reported not only with the anesthetic effect, but also significantly reduce hepatic ischemia–reperfusion injury [14]. Previous studies suggest that the mechanism of sevoflurane on protecting ischemia–reperfusion injury was related to a variety of physiological processes, such as reducing oxygen free radicals, inhibiting inflammatory reaction, reducing intracellular calcium overload as well as improving the energy metabolism of liver cells [15,16]. However, fewer studies have reported the relationship and potential mechanism between sevoflurane and Grp78 in hepatic ischemia–reperfusion injury.

Based on these existing researches, we speculated that Grp78 might be involved in hepatic ischemia–reperfusion and sevoflurane might be exerted its effects by affecting the expression Grp78. Therefore, we validated these speculations through in-depth research. The present study will provide an important theoretical basis for the application of sevoflurane in the prevention and treatment of hepatic ischemia–reperfusion injury.

## Methods

### Animals and grouping

A total of 24 male Sprague–Dawley rats (obtained from Laboratory Animal Center, Chinese Academy of Sciences, Shanghai) with an average weight of 240 ± 15 g were housed in a room at 20°C, 60% humidity. Each rat was kept individually in a cage and had free access to water and rat chow daily. After a week of feeding, these rats were randomly divided into three groups: Sham group, I/R group, and SEV group. In the present study, all animal experiments were approved by our ethics committee.

### Experiments

For rats of SEV group, they were pre-treated with sevoflurane at first. Briefly, rats were placed in a closed container communicating with the anesthesia machine. Sevoflurane evaporation tank was then opened to allow sevoflurane to flow through the closed container. Sevoflurane concentration was stabilized at 2.4% (1 minimum alveolar anesthetic concentration [MAC]) by using an anesthetic drug concentration monitor. Rats were kept breathing for 30 min under these conditions and then they were taken out from the closed container.

Rats of I/R group and SEV group were then given intraperitoneal injections of 1% pentobarbital (dose: 50 mg/kg). As we know, pentobarbital is an anesthetic drug commonly used before surgery. It has the advantages of rapid efficacy and long duration of anesthesia. Successful anesthesia standard was as follows: No limb reaction was occurred after rats were turned over. Rats were then fixed in the supine position on the operating table. In the middle of the abdomen, an incision was made to enter the abdominal cavity. The liver was then separated from its surrounding ligaments to expose the hilar. Non-destructive vessel clips were used to block the liver pedicles of left lobe and middle lobe (including the portal vein, hepatic artery, and bile duct). Liver ischemia was completed if the left lobe and middle lobe were gradually whitening. During the ischemia of the liver, the abdominal cavity was closed with a single layer of 4-0 silk suture, and 4 ml saline was injected subcutaneously to supplement the loss of intraoperative fluid. Non-destructive vessel clips were removed 2 h after liver ischemia to perform reperfusion by secondary laparotomy.

For rats of Sham group, after anesthesia, they were only underwent laparotomy and second laparotomy, while hepatic ischemia–reperfusion was not carried on them.

Two hours after reperfusion, vena cava blood samples of rats in each group were collected using syringes. After centrifugation at 4°C (12000 rev/min for 15 min), the supernatant was obtained and stored at −80°C. At last, liver samples were rapidly collected and washed with 0.9% saline. Total of 100 g liver samples were cut and prepared into 10% homogenate by using 0.9% saline, while the remaining samples were stored at −80°C.

### ELISA of serum TNF-α, IL-1β, IL-10, and IL-6 concentrations

The TNF-α, IL-1β, IL-10, and IL-6 levels in serum were determined by ELISA method and the testing process was carried out in strict accordance with the ELISA kit instructions (Invitrogen, U.S.A.).

### Determination of serum malondialdehyde, superoxide dismutase, and nitric oxide in liver homogenate

Homogenate (10%) was centrifuged at 2500 rev/min for 15 min to the obtain supernatant. The level of malondialdehyde (MDA), superoxide dismutase (SOD), and nitric oxide (NO) was determined by using detection kits. All kits were purchased from Nanjing Jiancheng Bioengineering Institute, China. The detection process was strictly performed according to the kit instructions.

### Hematoxylin–Eosin staining to detect pathological changes of liver tissue

The liver tissue samples from each group were fixed with 4% paraformaldehyde. After embedded in paraffin and sectioned, these tissues were underwent Hematoxylin–Eosin (HE) staining and their pathological changes were observed under light microscope.

### Tunel detection of liver tissue apoptosis

Tunel was performed to detect the apoptosis of liver tissue sections by using TUNEL kit (Invitrogen, U.S.A.) according to the instructions. Apoptotic cells were observed under optical microscope, and the nuclei of the positive cells were presented as brownish yellow. Five fields of each section were randomly selected to observe apoptosis.

### Immunohistochemical detection of Grp78 expression

Liver tissues were subjected to antigenic heat repair after being routinely dewaxed and hydrated. Then 3% H_2_O_2_–methanol was used to block endogenous peroxidase. Non-immune goat serum was used to block non-specific sites. Goat anti-human Grp78 was added for 15 min incubation and biotinylated antibody was used as secondary antibody for 10 min incubation. Diaminobenzidine (DAB) color reaction was performed before Hematoxylin counterstain. At last, these tissues were dehydrated using a gradient of alcohol, and were observed under optical microscope after being sealed with a neutral resin. The Grp78 protein was mainly expressed in the cytoplasm and on the cell membrane. The cytoplasm or membrane of Grp78 protein positive cells was appeared brown or yellowish brown.

### Construction of ischemia–reperfusion hepatocyte model

Mouse embryonic hepatocytes BNL CL.2 cells (Shanghai Institute of Biological Sciences, Chinese Academy of Sciences) were cultured in Dulbecco’s modified eagle medium (DMEM) medium with 10% FBS and kept in a 5% CO_2_ incubator at 37°C. Cells were collected in logarithmic growth phase and divided into three groups (Control group, I/R group, and SEV group) according to the different treatment methods. Cells of I/R group were subjected to establishment of ischemia–reperfusion cell model by oxygen–glucose deprivation. After these cells were washed twice with phosphate buffered solution (PBS), they were prepared as a cell suspension by Kreb solution (composed of 119 mmol/l NaCl, 4.7 mmol/l KCl, 1.2 mmol/l K_2_H_2_PO_4_, 25 mmol/l NaHCO_3_, 2.5 mmol/l CaCl, 1 mmol/l MgCl, pH = 7.3) and were seeded in a 6-well plate at a density of 1 × 10^5^ ml^−1^ with 3 ml each well. After incubated for 4 h in an anoxic tank, the residual Kreb solution was discarded and DMEM medium (10% FBS) was used to resuspend these cells for additional 12 h incubation in the 5% CO_2_ incubator at 37°C. The same process of ischemia–reperfusion was performed in cells of SEV group. However, it should be noted that, before ischemic treatment, cells of SEV group were placed in a semi-airtight container and 2% sevoflurane was used for 2 h of pre-treatment. For cells of Control group, they were dispersed into cell suspensions with a density of 1 × 10^5^ ml^−1^ by using DMEM (10% FBS) and were seeded in a 6-well plate with 3 ml of cell suspension per well. Then they were kept in a 5% CO_2_ incubator at 37°C for 16 h.

### Cells transfection and grouping

Normal BNL CL.2 cells were collected and transfected by Grp78 siRNA or negative control Grp78 siRNA. They were named as siGrp78 group and siNC group, respectively. In addition, for cells of I/R group, they were also transfected by negative control Grp78 siRNA or Grp78 siRNA and were set as I/R + siNC group or I/R + siGrp78 group, respectively. Meanwhile, cells of SEV group were also collected and transfected by negative control Grp78 siRNA. They were renamed as I/R + siNC + SEV group in this section. They were underwent transfection by Grp78 siRNA and were set as I/R + siGrp78 + SEV group. Lipofectamine 2000 (Invitrogen, U.S.A.) was used to perform transfection according to the instructions. DMEM (10% FBS) medium was used to suspend successfully transfected cells of each group. These cells were then seeded in 6-well plates at a density of 1 × 10^4^ ml^−1^ respectively for 48 h incubation.

### Apoptosis detection by flow cytometry

Cells of each group were collected and centrifugation was carried out at 1000 rev/min for 5 min. After removing the supernatant, the cell pellet on the bottom was washed twice with PBS. Then 10 μl of PI (50 μg/ml) as well as 10 μl of RNaseA (100 μg/ml) was added into these cells for 30 min incubation at 4°C in darkness. Apoptosis was detected using flow cytometry.

### Quantitative real-time PCR assay

After 48 h incubation, cells of siGrp78 group and siNC group were collected and total RNA in cells was extracted by TRIzol (Invitrogen, U.S.A.). After obtaining cDNA template by reverse transcription reaction (Grp78 forward primer 5′-GACATCAAGTTCTTGCCGTT-3′ and reverse primer 5′-CTCATAACATTTAGGCCAGC-3′; GAPDH forward primer 5′-CGTATTGGGCGCCTGGTCACC-3′ and reverse primer 5′-GGGATGATGTTCTGGAGAGCCC-3′). PCR amplification reaction containing 36 cycles was carried out with the following procedures: degeneration for 10 s at 95°C, reannealing for 20 s at 58°C, and extension for 34 s at 72°C. In the present study, 2-ΔΔCt method was used to process data.

### Western blotting analysis

Cell total protein of each group was extracted by RIPA lysis buffer and BCA protein assay kit (Beyotime, Shanghai, China) was used for protein concentration detection. After electrophoresis with 10% sodium dodecyl sulfate polyacrylamide gel electrophoresis (SDS-PAGE), protein was transferred onto a polyvinylidene fluoride (PVDF) membrane. In the present study, protein blocking was performed for 2 h by using skimmed milk (5%). Mouse anti-rabbit primary antibody (1:1000) was added for 12 h incubation at 4°C. Then goat anti-mouse secondary antibody (1:5000) was used for additional 1 h incubation at room temperature. After the membrane was washed three times with TBST, color reaction and data analysis were carried out. GAPDH was selected as internal reference.

### Statistical analysis

Data were expressed as the mean ± standard deviation and processed by SPSS 17.0 and GraphPad Prism 5.0. Comparison between two groups was tested using *t*-test while comparison among multiple groups was performed using ANOVA. *P*<0.05 was considered as statistically significant. All data were repeated at least three independent experiments.

## Results

### Sevoflurane reduced liver damage degree, liver cell apoptosis, and Grp78 expression

**Figure 1:**
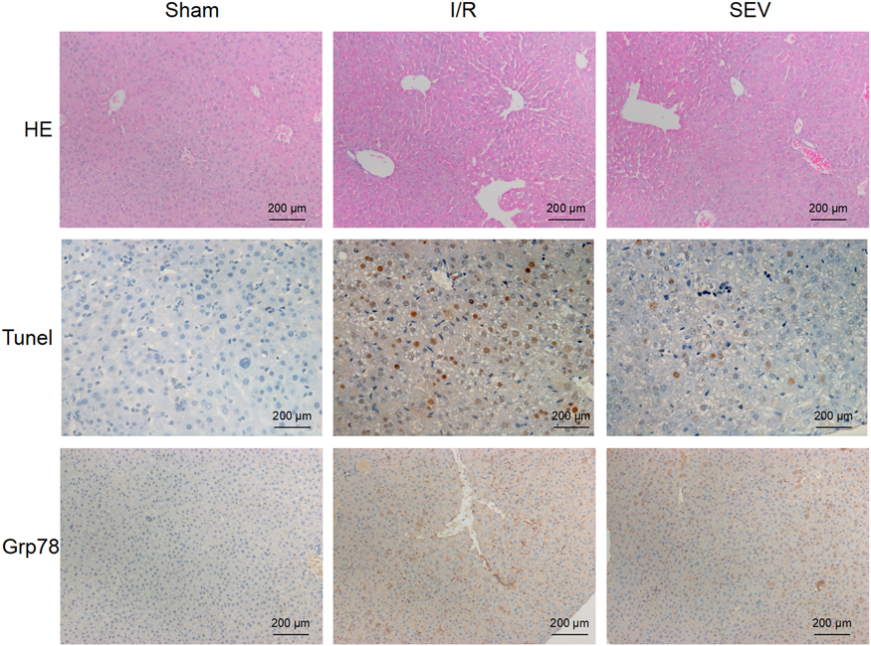
Sevoflurane reduced liver damage degree, liver cell apoptosis, and Grp78 expression Detection of liver damage degree by HE staining, detection of liver cell apoptosis by Tunel, and detection of Grp78 expression by immunohistochemistry.

HE staining results showed that, for rats of Sham group, cells of liver tissue were arranged in an orderly manner and normal cell morphology was found. However, compared with Sham group, serious liver damage was found in rats liver tissue of I/R group, such as irregular cell arrangement and hepatocyte balloon degeneration. While obviously reduced liver damage degree was occurred in rats of SEV group when compared with that of I/R group. In addition, we also found from Tunel detection that the number of apoptotic cells in rats liver tissue of I/R group was significantly higher than that of Sham group. At the same time, apoptotic cell number of SEV group was still higher than that of Sham group, but which was much lower when compared with that of I/R group. Furthermore, according to immunohistochemistry results, the number of Grp78 positive cells of I/R group was the highest, followed by SEV group. Rats liver tissue of Sham group was with the lowest number of protein positive cells (Figure 1). The above results revealed that sevoflurane could reduce liver damage degree, liver cell apoptosis, and Grp78 expression of liver ischemia–reperfusion rats.

### Sevoflurane affected inflammatory factor levels, and MDA, SOD, and NO

**Figure 2:**
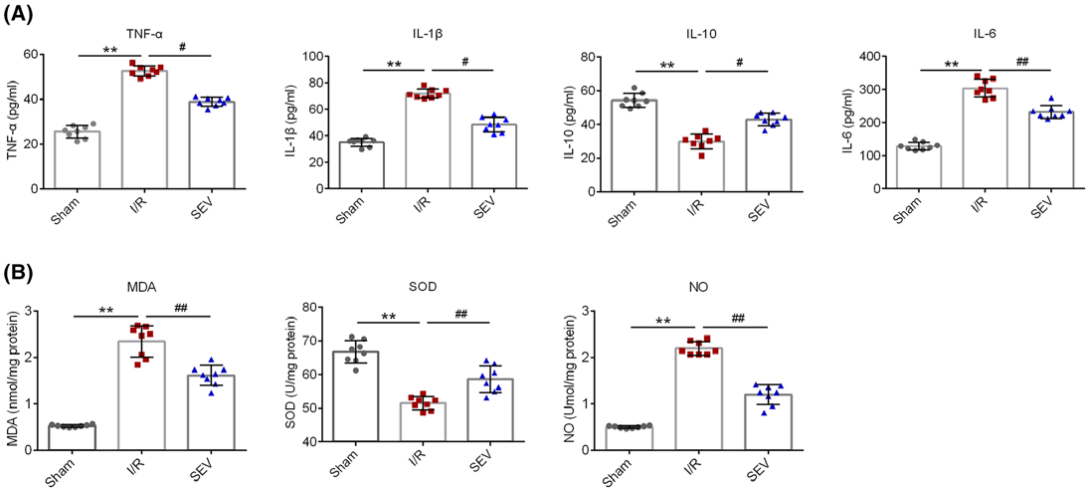
Concentrations of TNF-α, IL-1β, IL-10, IL-6, MDA, SOD, and NO (**A**) Detection of TNF-α, IL-1β, IL-10, and IL-6 concentrations by ELISA; (**B**) detection of MDA, SOD, and NO concentrations. ** *P*<0.01 when compared with Sham group; ^#^*P*<0.05 or ^##^*P*<0.01 when compared with I/R group.

For rats of I/R group, TNF-α, IL-1β, and IL-6 concentrations were significantly increased (*P*<0.01) while IL-10 concentration was significantly decreased (*P*<0.01) when compared with Sham group. However, compared with I/R group, obviously decreased TNF-α, IL-1β, and IL-6 concentrations and much increased IL-10 concentration were found in rats of SEV group (*P*<0.05 or *P*<0.01) (Figure 2A). In addition, we also observed significantly increased MDA and NO concentrations (*P*<0.01) as well as significantly decreased SOD concentration (*P*<0.01) in I/R group compared with those of Sham group. But after sevoflurane treatment, MDA and NO concentrations were dramatically lower (*P*<0.01) and SOD concentration was significantly higher (*P*<0.01) in rats of SEV group when compared with those of I/R group (Figure 2B). These results demonstrated that sevoflurane could inhibit TNF-α, IL-1β, IL-6 levels and increase IL-10 level. It also could reduce MDA and NO levels and increase SOD level.

### Sevoflurane reduced BNL CL.2 cells apoptosis and expression of Grp78, PERK, eIF2α, p-c-JNK/JNK after ischemia–reperfusion

**Figure 3:**
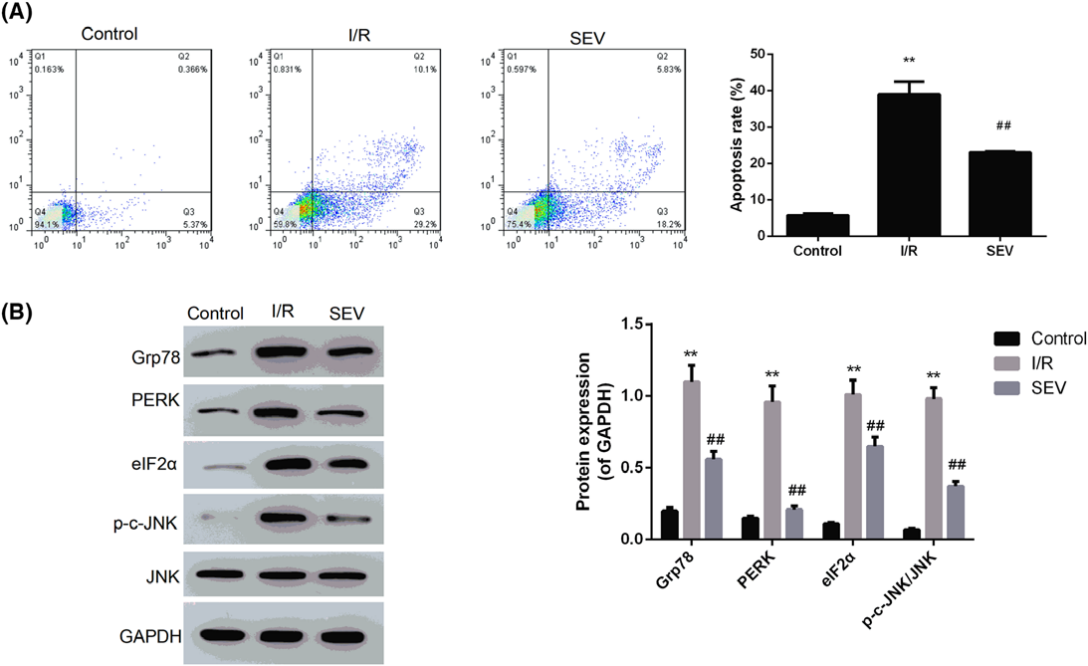
Sevoflurane reduced BNL CL.2 cells apoptosis and expression of Grp78, PERK, eIF2α, p-c-JNK/JNK after ischemia–reperfusion (**A**) Detection of apoptosis by flow cytometry; (**B**) detection of Grp78, PERK, eIF2α, p-c-JNK, and JNK expression by Western blotting analysis. The molecular weights for protein blots were as follows: Grp78, 72 kDa; PERK, 125 kDa; eIF2α, 65 kDa; p-c-JNK, 46–54 kDa; JNK, 48 kDa; GAPDH, 36 kDa. ** *P*<0.01 when compared with Control group; ^##^*P*<0.01 when compared with I/R group.

Apoptosis detection by flow cytometry showed that, compared with Control group, apoptosis rate of I/R group was dramatically higher (*P*<0.01). Although apoptosis rate of SEV group was still higher than that of Control group, significantly decreased apoptosis rate was also found when it was compared to I/R group (*P*<0.01), illustrating that sevoflurane pre-treatment could significantly reduce cell apoptosis caused by ischemia–reperfusion (Figure 3A). Furthermore, Western blotting analysis also revealed that expression of JNK in cells of the three groups was not significantly different. However, Grp78, PERK, eIF2α, and p-c-JNK expression in I/R group was significantly higher than those in Control group (*P*<0.01). In addition, when compared with I/R group, the expression of Grp78, PERK, eIF2α, and p-c-JNK in SEV group was dramatically decreased (*P*<0.01) (Figure 3B). It was suggested that sevoflurane pre-treatment had no effect on the expression of JNK, but significantly reduced the expression of Grp78, PERK, eIF2α, and p-c-JNK.

### Sevoflurane reduced apoptosis and expression of PERK, eIF2α, p-c-JNK/JNK by inhibiting the expression of Grp78

**Figure 4:**
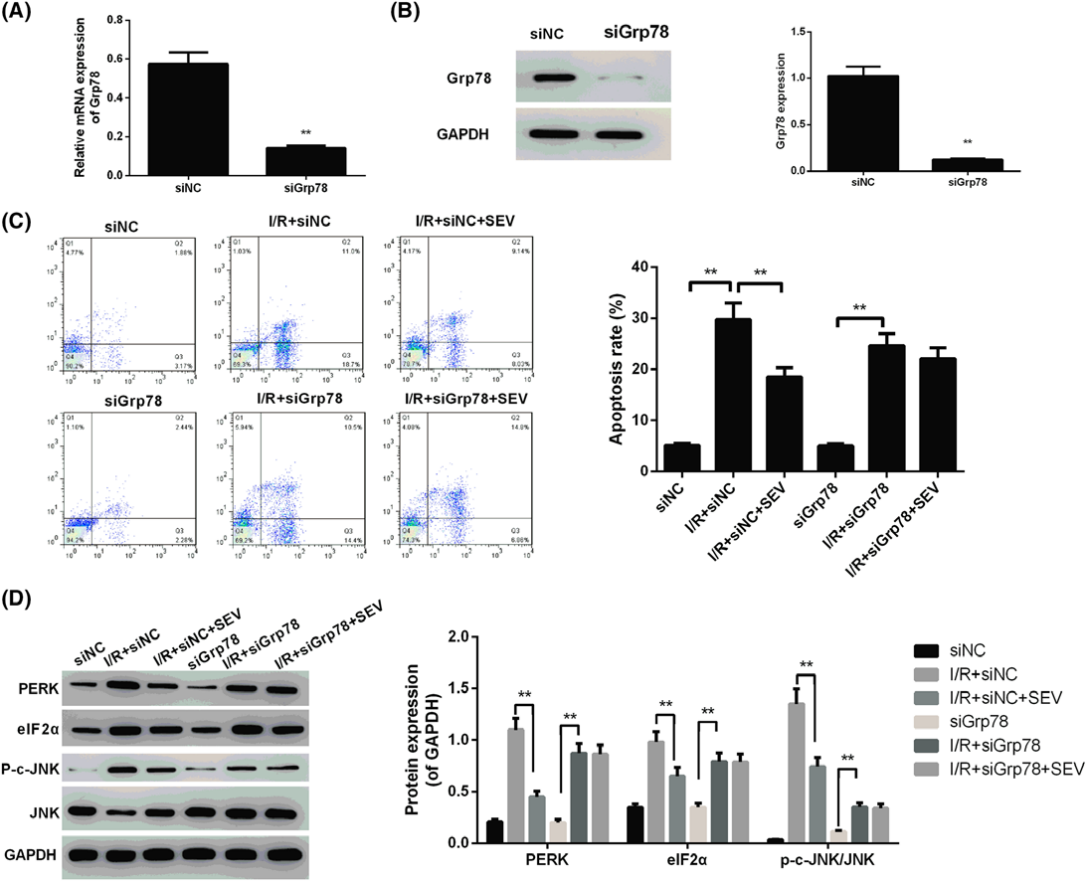
Sevoflurane reduced apoptosis and expression of PERK, eIF2α, p-c-JNK/JNK by inhibiting the expression of Grp78 (**A**) Detection of Grp78 mRNA by qTR-PCR; (**B**) detection of Grp78 protein expression by Western blotting analysis; (**C**) detection of apoptosis by flow cytometry; (**D**) detection of PERK, eIF2α, JNK, and p-c-JNK expression by Western blotting analysis. The molecular weights for protein blots were as follows: Grp78, 72 kDa; PERK, 125 kDa; eIF2α, 36 kDa; p-c-JNK, 46-54 kDa; JNK, 48 kDa; GAPDH, 36 kDa. ** *P*<0.01.

After being transfected, siGrp78 mRNA and protein expression in BNL CL.2 cells of siGrp78 group was significantly lower than that in siNC group (*P*<0.01) (Figure 4A,B). We also noted that, for cells of siNC group, I/R + siNC group and I/R + siNC + SEV group, the apoptosis rate of I/R + siNC group was significantly higher than that of siNC group (*P*<0.01). Cells of I/R + siNC + SEV group were with much lower apoptosis rate when compared with I/R + siNC group (*P*<0.01). In addition, when compared with siGrp78 group, significantly increased apoptosis rate was found in I/R + siGrp78 group (*P*<0.01). However, no significant difference in apoptosis rate was found between I/R + siGrp78 group and I/R + siGrp78 + SEV group (Figure 4C).

Furthermore, Western blotting analysis revealed that significantly higher PERK, eIF2α, and 6p-c-JNK/JNK protein relative expression was found in I/R + siNC group when compared with siNC group (*P*<0.01). But these protein relative expression in I/R + siNC + SEV group was all markedly lower than those of I/R + siNC group (*P*<0.01). Meanwhile, it could also be noticed that PERK, eIF2α, and p-c-JNK/JNK protein relative expression in I/R + siGrp78 group was much higher than those of siGrp78 group (*P*<0.01). However, when compared with I/R + siGrp78 group, changes of these protein relative expression in I/R + siGrp78 + SEV group were not obvious (Figure 4D). These results suggested that the inhibitory effect of sevoflurane on ischemia–reperfusion BNL CL.2 cells apoptosis and expression of PERK, eIF2α, p-c-JNK/JNK proteins was through inhibiting the expression of Grp78.

## Discussion

In the present study, we explored the effects of sevoflurane pre-conditioning on hepatic ischemia–reperfusion injury in rats, and further investigated its effect on ischemia–reperfusion hepatocytes *in vitro* by using BNL CL.2 cells. The results demonstrated that sevoflurane pre-conditioning could reduce the degree of ischemia–reperfusion liver damage and the level of serum inflammatory cytokines. Further *in vitro* studies also revealed that sevoflurane pre-conditioning could reduce hepatocyte apoptosis and PERK, eIF2α, and p-c-JNK expression by inhibiting Grp78 expression.

Hepatic ischemia–reperfusion injury has serious adverse effect on patients prognosis [17]. Some studies have shown that cytokines are involved in the entire pathophysiological process of hepatic ischemia–reperfusion injury through aggravating inflammatory reactions and cell damage [18–20]. Interaction of these inflammatory factors exacerbate inflammatory response, leading to the recruitment and infiltration of leukocytes in the liver and thereby aggravating microcirculation disorders [21,22]. Many studies have shown that up-regulation of TNF-α, IL-1β, and IL-6 could directly lead to organ damage by aggravating inflammatory response, and IL-10 could exert an inhibitory effect on inflammation [23,24]. Our results further confirmed these previous studies that significantly declined serum TNF-α, IL-1β, and IL-6 levels was occurred and serum IL-10 level was increased in hepatic ischemia–reperfusion rats by sevoflurane pre-conditioning, which effectively relieved hepatic ischemia–reperfusion injury. In addition, we also observed that sevoflurane significantly reduced the apoptosis of liver tissue cells. Apoptosis is a common phenomenon in the process of hepatic ischemia–reperfusion [25,26]. It has been reported that 50–70% of endothelial cells and 40–60% of hepatocytes underwent apoptosis during hepatic ischemia–reperfusion [27]. Effective blocking of hepatic cell apoptosis signal transduction pathway is one of the important ways to reduce hepatocytes damage and maintain hepatic function. Our study not only proved that sevoflurane pre-conditioning could effectively inhibit cell apoptosis, but also demonstrated that sevoflurane could inhibit the expression of Grp78, PERK, eIF2α, and p-c-JNK. The relevant mechanism was further conducted by *in vitro* cell studies and we found that sevoflurane could reduce hepatocyte apoptosis and PERK, eIF2α, and p-c-JNK expression by inhibiting Grp78 expression.

Grp78 was reported to regulate endoplasmic reticulum homeostasis as well as cell stress response [28,29]. Studies have shown that ischemia–reperfusion injury could lead to an imbalance of the endoplasmic reticulum, thereby inducing endoplasmic reticulum stress [30,31]. Grp78 is localized in the endoplasmic reticulum [32]. Severe ischemia–reperfusion injury would significantly up-regulate the expression of Grp78 [33,34]. It can start the endoplasmic reticulum endogenous protective mechanisms to maintain the balance of intracellular homeostasis [29]. In this research, we also found that Grp78 protein expression was significantly up-regulated in rat liver tissues or cells following ischemia–reperfusion. Down-regulation of Grp78 was occurred because of relieved liver tissue or cell damage by sevoflurane pre-treatment. PERK, eIF2α, and p-c-JNK expression levels were also declined with the down-regulation of Grp78. PERK is a serine threonate kinase, which can be activated upon endoplasmic reticulum stress activation [35,36]. Endoplasmic reticulum stress will be activated if the cell apoptosis and necrosis was occurred [37,38]. As one of the endoplasmic reticulum kinases, PERK is also activated. eIF2α and p-c-JNK expression was reported to be involved in apoptosis and many researches have proved that eIF2α and p-c-JNK had inhibitory effect on a variety of cancer cells apoptosis [39–41]. In the present study, sevoflurane attenuated the apoptosis of cells after hepatic ischemia–reperfusion and led to the down-regulation of Grp78, which further resulted in the decline of PERK, eIF2α, and p-c-JNK expression.

This article has some limitations. Hepatic ischemia and reperfusion could be better reflected by Doppler ultrasound. However, we could not provide this data due to the limitations of laboratory conditions. In our future research, we will conduct further researches on this point if laboratory conditions permit.

In conclusion, this article researched the effects of sevoflurane on hepatic ischemia–reperfusion injury through *in vivo* and *in vitro* studies. The results suggested that sevoflurane pre-treatment could reduce degree of ischemia–reperfusion liver damage and inhibit the apoptosis of liver cells. The mechanism involved in this process was that sevoflurane could inhibit hepatic ischemia–reperfusion injury by inhibiting the expression of Grp78. The present study provided a potential target for the targetted treatment of hepatic ischemia–reperfusion injury.

## Abbreviations

DMEM: Dulbecco’s modified eagle medium
Grp78: glucose regulatory protein 78
HE: Hematoxylin–Eosin
MDA: malondialdehyde
PBS: phosphate buffered solution
SEV: sevoflurane
SOD: superoxide dismutase
TBST: Tris-buffered saline Tween
